# 4-Hy­droxy-1,2,6-tri­methyl­pyridinium chloride monohydrate

**DOI:** 10.1107/S1600536813011616

**Published:** 2013-05-04

**Authors:** T. Seethalakshmi, S. Manivannan, S. Dhanuskodi, Daniel E. Lynch, S. Thamotharan

**Affiliations:** a Department of Physics, Government Arts College (Autonomous), Karur 639 005, India; b Carbon Nanomaterials Laboratory, Department of Physics, National Institute of Technology, Tiruchirappalli 620 015, India; c School of Physics, Bharathidasan University, Tiruchirappalli 620 024, India; d Faculty of Health and Life Sciences, Coventry University, Coventry CV1 5FB, England; e Department of Bioinformatics, School of Chemical and Biotechnology, SASTRA University, Thanjavur 613 401, India

## Abstract

In the crystal of the title hydrated mol­ecular salt, C_8_H_12_NO^+^·Cl^−^·H_2_O, the water mol­ecule makes two O—H⋯Cl hydrogen bonds, generating [010] zigzag chains of alternating water mol­ecules and chloride ions. The cation is bonded to the chain by an O—H⋯O hydrogen bond and two weak C—H⋯Cl inter­actions. Weak aromatic π–π stacking [centroid–centroid separation = 3.5175 (15) Å] occurs between the chains.

## Related literature

For related structures, see: Seethalakshmi *et al.* (2006*a* ▶,*b* ▶,*c* ▶, 2007 ▶). For related compounds, see: Dhanuskodi *et al.* (2006 ▶, 2008 ▶).
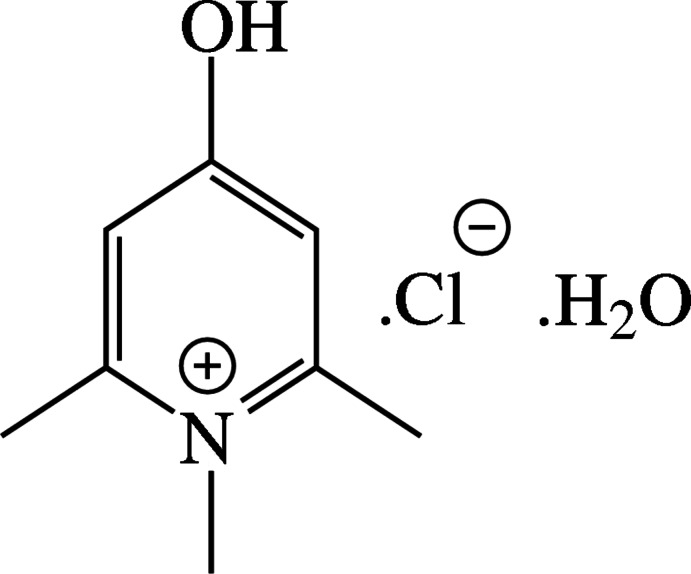


## Experimental

### 

#### Crystal data

C_8_H_12_NO^+^·Cl^−^·H_2_O
*M* *_r_* = 191.65
Monoclinic, 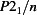

*a* = 8.2548 (11) Å
*b* = 8.4781 (9) Å
*c* = 13.6714 (18) Å
β = 99.064 (6)°
*V* = 944.8 (2) Å^3^
*Z* = 4
Mo *K*α radiation
μ = 0.37 mm^−1^
*T* = 120 K
0.54 × 0.42 × 0.16 mm

#### Data collection

Bruker–Nonius 95mm CCD camera on κ-goniostat diffractometer
Absorption correction: multi-scan (*SADABS*; Sheldrick, 2003 ▶) *T*_min_ = 0.827, *T*_max_ = 0.944
9882 measured reflections
2159 independent reflections
1546 reflections with *I* > 2σ(*I*)
*R*_int_ = 0.069

#### Refinement

*R*[*F* ^2^ > 2σ(*F* ^2^)] = 0.061
*wR*(*F* ^2^) = 0.174
*S* = 1.02
2159 reflections
124 parameters
2 restraints
H atoms treated by a mixture of independent and constrained refinement
Δρ_max_ = 0.73 e Å^−3^
Δρ_min_ = −0.48 e Å^−3^

### 

Data collection: *COLLECT* (Nonius, 1998 ▶); cell refinement: *DENZO* (Otwinowski & Minor, 1997 ▶); data reduction: *DENZO*; program(s) used to solve structure: *SIR92* (Altomare *et al.*, 1994 ▶); program(s) used to refine structure: *SHELXL97* (Sheldrick, 2008 ▶); molecular graphics: *ORTEP-3 for Windows* (Farrugia, 2012 ▶) and *PLATON* (Spek, 2009 ▶); software used to prepare material for publication: *SHELXL97*.

## Supplementary Material

Crystal structure: contains datablock(s) I, global. DOI: 10.1107/S1600536813011616/hb7075sup1.cif

Structure factors: contains datablock(s) I. DOI: 10.1107/S1600536813011616/hb7075Isup2.hkl

Supplementary material file. DOI: 10.1107/S1600536813011616/hb7075Isup3.cml

Additional supplementary materials:  crystallographic information; 3D view; checkCIF report

## Figures and Tables

**Table 1:** Hydrogen-bond geometry (Å, °)

*D*—H⋯*A*	*D*—H	H⋯*A*	*D*⋯*A*	*D*—H⋯*A*
O1—H1⋯O1*W* ^i^	0.82 (4)	1.78 (4)	2.591 (3)	168 (4)
O1*W*—H1*W*⋯Cl1	0.81 (2)	2.31 (2)	3.095 (2)	162 (4)
O1*W*—H2*W*⋯Cl1^ii^	0.82 (2)	2.30 (2)	3.106 (2)	168 (4)
C3—H3⋯Cl1^i^	0.95	2.72	3.647 (3)	165
C9—H9*A*⋯Cl1^iii^	0.98	2.80	3.704 (3)	154

Symmetry codes: (i) 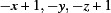; (ii) 
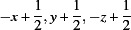
; (iii) 
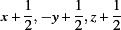
.

**Fig. 1.**
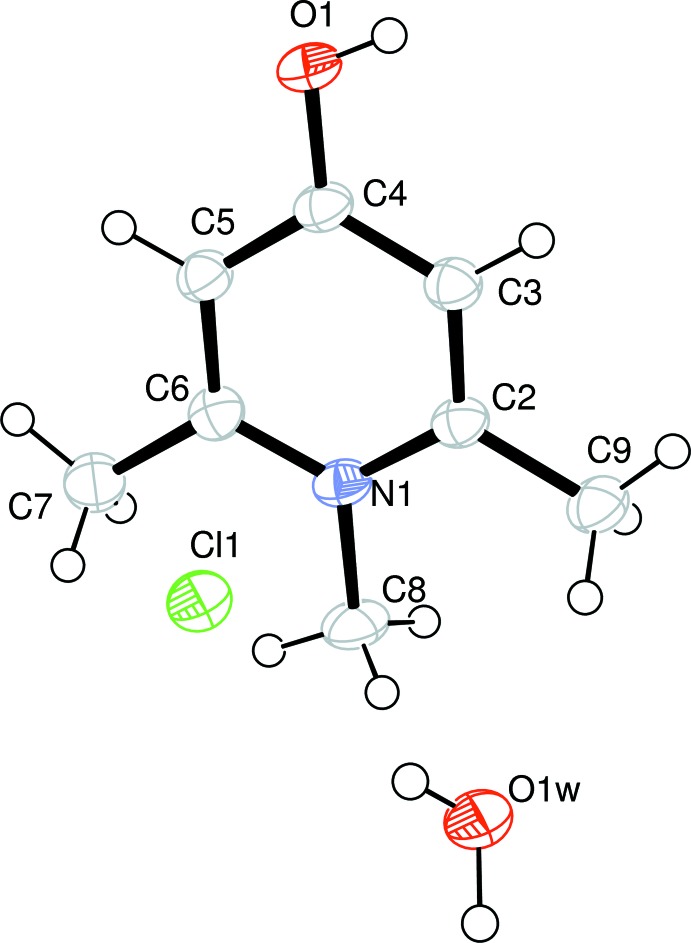
Molecular structure of (I), showing displacement ellipsoids drawn at the 50% probability level.

**Fig. 2.**
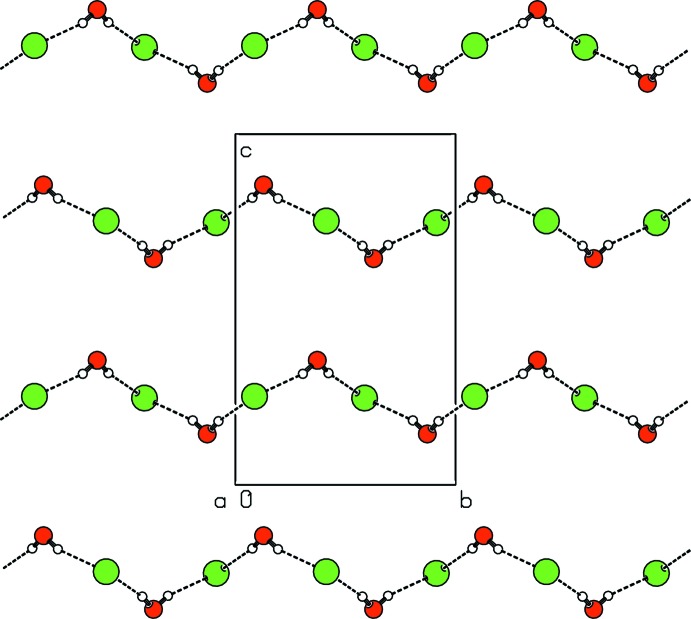
One dimensional chain generated from alternate water and chloride anion interconnected by O—H···Cl hydrogen bond which runs parallel to the b axis.

**Fig. 3.**
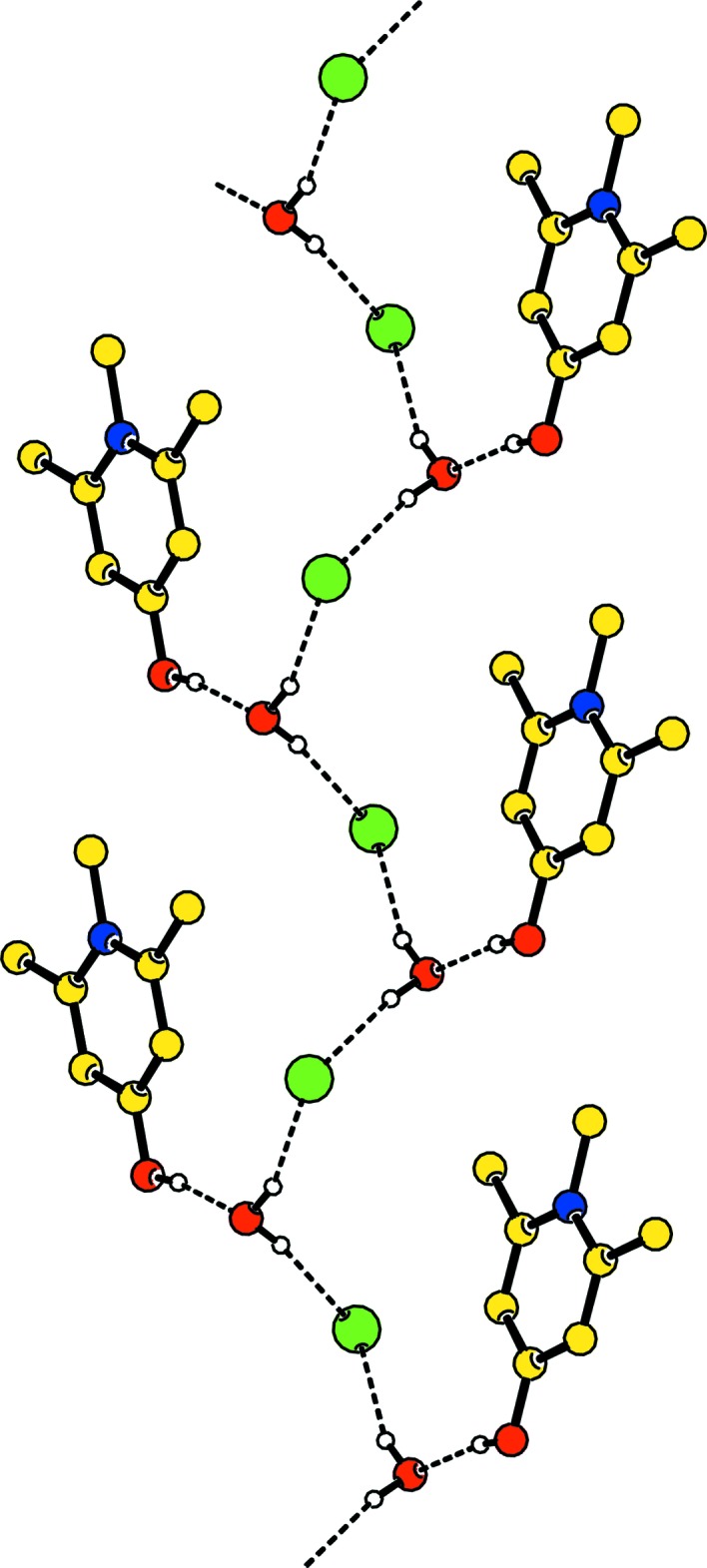
Part of the crystal structure showing O—H···O—H···Cl···H—O···H—O and O—H···O—H···Cl···H—O—H···Cl···H—O···H—O cooperative hydrogen bonding modes interconnects two cations related by translation and glide, respectively. For clarity, H atoms not involved in the hydrogen bonds have been omitted.

**Fig. 4.**
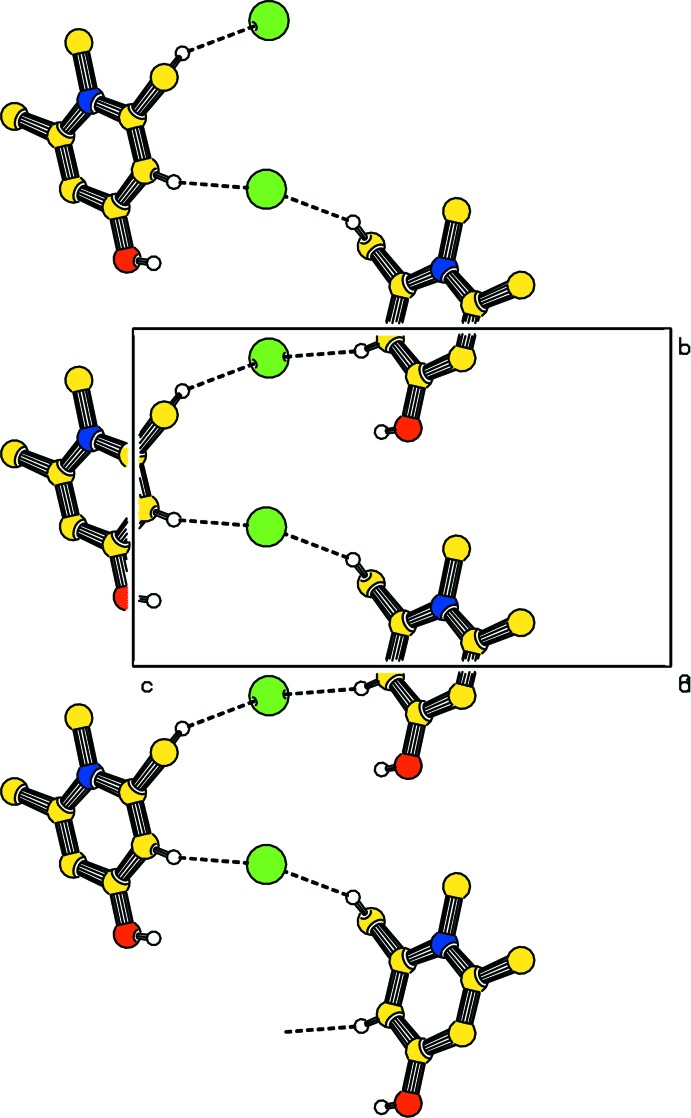
Part of the crystal structure showing a helical chain formed by C—H···Cl intermolecular interactions.

**Fig. 5.**
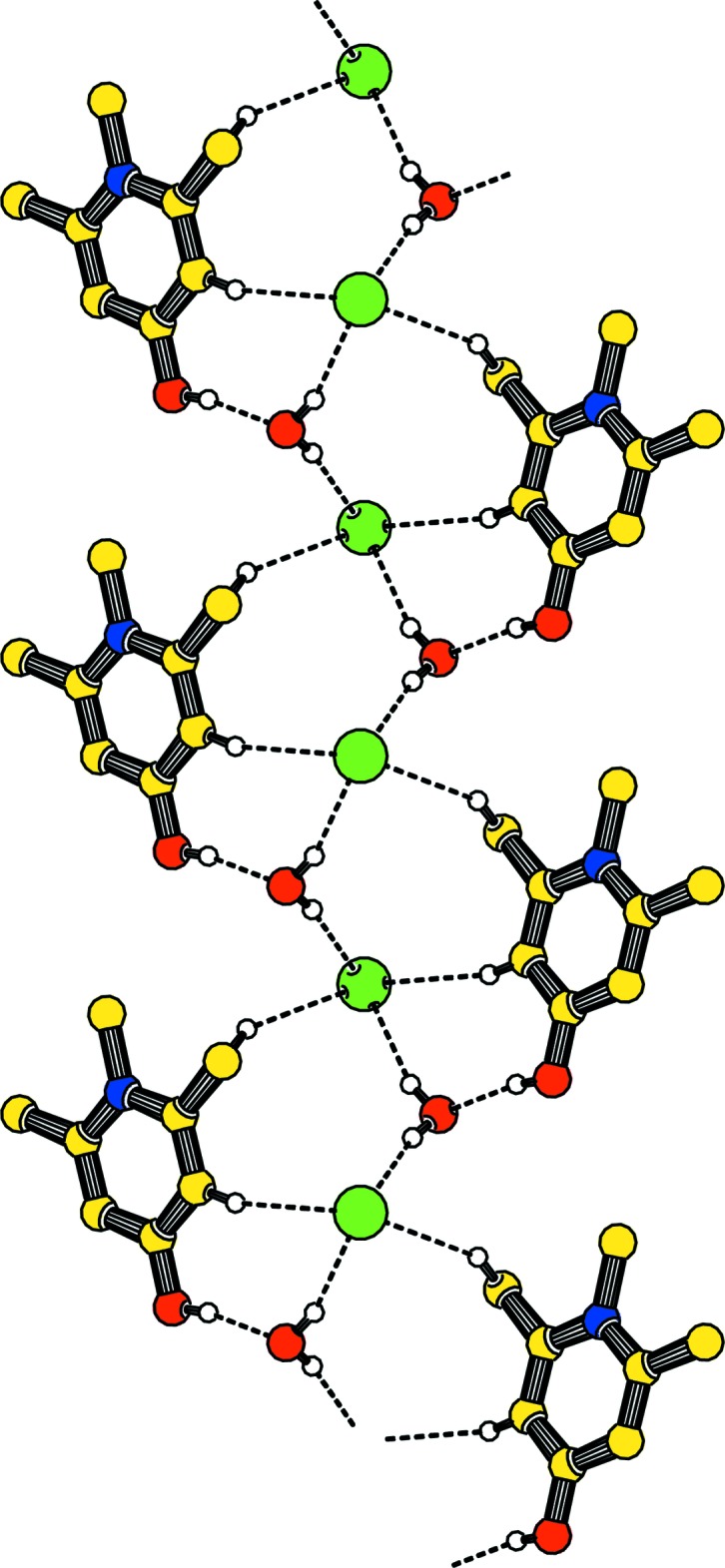
Arrangement of alternate R23(8) and R24(10) ring motifs.

## supplementary crystallographic information

### Comment

As part of our ongoing studies on 4-hydroxypyridinum salts (Seethalakshmi *et al.*, 2007, Dhanuskodi *et al.*, 2006, 2008), we report here the crystal structure of *N*-methyl-2,6-dimethyl-4-hydroxypyridinium chloride monohydrate (I), (Fig. 1).

The corresponding bond lengths and angles of the cation in (I) are comparable with those of related structures reported earlier (Seethalakshmi *et al.*, 2006*a*,*b*,*c*, 2007).

In (I), water molecule acts as a donor for two different symmetry-related chloride anions and acts as an acceptor for the hydroxy group of the cation. As shown in Fig.2, the water molecule and chloride anion are interlinked by O—H···Cl intermolecular hydrogen bond. This interaction links the water molecule and the chloride anion alternately into a one-dimensional chain which runs paralell to the *b* axis. The cation molecules in the crystal structure are interlinked *via* two types of cooperative hydrogen bonding modes (Fig. 3). For example, the glide related cation molecules are interconnected by O—H···O—H···Cl···H—O···H—O cooperative hydrogen bonding pattern, whereas cations are related by translation interlinked *via* another type of O—H···O—H···Cl···H—O—H···Cl···H—O···H—O cooperative hydrogen bonding pattern.

The title salt (I), also features a network of weak intermolecular C—H···Cl interactions. Atoms C3 (*via* H3) and C9 (*via* H9A) of cation are involved in weak C—H···Cl intermolecular interactions with two different chloride anions. These weak intermolecular interactions link the cations through chloride anions and generates a helical chain which runs paralell to *b* axis. The chloride anions are located approximately at the middle of the helical axis (Fig. 4).

As shown in Fig. 5, an *R*^2^_3_(8) loop is formed by the combination of O—H···O1W and O1W—H1W···Cl and C3—H3···Cl intermolecular interactions. As mentioned earlier, one of the methyl atoms C9 (*via* H9A) is participated in a weak intermolecular C—H···Cl interaction with chloride anion. Again, this interaction combines with C3—H3···Cl and two O1w—H···Cl interactions forming a ring which has a graph-set motif of *R*^2^_4_(10). The *R*^2^_3_(8) and *R*^2^_4_(10) ring motifs are arranged alternately as a helical ribbon which run parallel to the *b* axis (Fig. 5). In the solid state, each chloride anion is tetra coordinated by two cations (*via* H3 and H9A) and two water molecules (*via* H1W and H2W). The tetra coordination angles in the range of 58.40–88.17°. There is a π···π stacking interaction also observed between two pyridinium rings related by center of inversion with centroid-to-centroid distance of 3.5175 (15) Å.

### Experimental

The title salt was prepared by dissolving 1-methyl-2,6-dimethyl-4-hydroxypyridine (1.37 g) with HCl (0.92 ml) in distilled water (5 ml). The mixture was stirred at room temperature for 7 h and the clear solution was kept for evaporation at 60 °C after filtration. Finally crystalline powder was obtained and dissolved in distilled water. Colourless prisms were obtained following the slow evoporation technique.

### Refinement

The positions of hydroxy H atom and H atoms of water molecule were determined from a difference Fourier map and refined freely along with their isotropic displacement parameters. In the final round of refinement, the O—H bond lengths of water molecule are restrained to 0.84 (2) Å. The methyl H atoms were constrained to an ideal geometry (C—H = 0.98 Å), with *U*_iso_(H) = 1.5*U*_eq_(C), but were allowed to rotate freely about the C—C and N—C bonds. The remaining H atoms were placed in geometrically idealized positions (C—H = 0.95 Å), with *U*_iso_(H) = 1.2*U*_eq_(C) and were constrained to ride on their parent atoms.

### Crystal data

**Table:**

C_8_H_12_NO^+^·Cl^−^·H_2_O	*F*(000) = 408
*M**_r_* = 191.65	*D*_x_ = 1.347 Mg m^−^^3^
Monoclinic, *P*2_1_/*n*	Mo *K*α radiation, λ = 0.71073 Å
Hall symbol: -P 2yn	Cell parameters from 1861 reflections
*a* = 8.2548 (11) Å	θ = 1.0–27.5°
*b* = 8.4781 (9) Å	µ = 0.37 mm^−^^1^
*c* = 13.6714 (18) Å	*T* = 120 K
β = 99.064 (6)°	Prism, colourless
*V* = 944.8 (2) Å^3^	0.54 × 0.42 × 0.16 mm
*Z* = 4	

### Data collection

**Table:**

Bruker–Nonius 95mm CCD camera on κ-goniostat diffractometer	2159 independent reflections
Radiation source: Bruker-Nonius FR591 rotating anode	1546 reflections with *I* > 2σ(*I*)
Graphite monochromator	*R*_int_ = 0.069
Detector resolution: 9.091 pixels mm^-1^	θ_max_ = 27.5°, θ_min_ = 2.8°
φ and ω scans	*h* = −10→10
Absorption correction: multi-scan (*SADABS*; Sheldrick, 2003)	*k* = −10→11
*T*_min_ = 0.827, *T*_max_ = 0.944	*l* = −17→17
9882 measured reflections	

### Refinement

**Table:**

Refinement on *F*^2^	Primary atom site location: structure-invariant direct methods
Least-squares matrix: full	Secondary atom site location: difference Fourier map
*R*[*F*^2^ > 2σ(*F*^2^)] = 0.061	Hydrogen site location: inferred from neighbouring sites
*wR*(*F*^2^) = 0.174	H atoms treated by a mixture of independent and constrained refinement
*S* = 1.02	*w* = 1/[σ^2^(*F*_o_^2^) + (0.1015*P*)^2^ + 0.5878*P*] where *P* = (*F*_o_^2^ + 2*F*_c_^2^)/3
2159 reflections	(Δ/σ)_max_ < 0.001
124 parameters	Δρ_max_ = 0.73 e Å^−^^3^
2 restraints	Δρ_min_ = −0.48 e Å^−^^3^

### Special details

**Table:**

Experimental. The minimum and maximum absorption values stated above are those calculated in *SHELXL97* from the given crystal dimensions. The ratio of minimum to maximum apparent transmission was determined experimentally as 0.597412.
Geometry. All e.s.d.'s (except the e.s.d. in the dihedral angle between two l.s. planes) are estimated using the full covariance matrix. The cell e.s.d.'s are taken into account individually in the estimation of e.s.d.'s in distances, angles and torsion angles; correlations between e.s.d.'s in cell parameters are only used when they are defined by crystal symmetry. An approximate (isotropic) treatment of cell e.s.d.'s is used for estimating e.s.d.'s involving l.s. planes.
Refinement. Refinement of *F*^2^ against ALL reflections. The weighted *R*-factor *wR* and goodness of fit *S* are based on *F*^2^, conventional *R*-factors *R* are based on *F*, with *F* set to zero for negative *F*^2^. The threshold expression of *F*^2^ > σ(*F*^2^) is used only for calculating *R*-factors(gt) *etc*. and is not relevant to the choice of reflections for refinement. *R*-factors based on *F*^2^ are statistically about twice as large as those based on *F*, and *R*- factors based on ALL data will be even larger.

### Fractional atomic coordinates and isotropic or equivalent isotropic displacement parameters (Å^2^)

**Table:**

	*x*	*y*	*z*	*U*_iso_*/*U*_eq_	
Cl1	0.45702 (8)	0.08706 (8)	0.25244 (5)	0.0322 (3)	
O1	0.8028 (3)	−0.2941 (2)	0.48865 (16)	0.0335 (5)	
O1W	0.3198 (3)	0.3719 (2)	0.35499 (17)	0.0347 (5)	
N1	0.7943 (3)	0.1762 (3)	0.42120 (16)	0.0267 (5)	
C2	0.7200 (3)	0.1258 (3)	0.49853 (19)	0.0261 (6)	
C3	0.7204 (3)	−0.0310 (3)	0.5220 (2)	0.0275 (6)	
H3	0.6686	−0.0657	0.5755	0.033*	
C4	0.7963 (3)	−0.1405 (3)	0.4678 (2)	0.0269 (6)	
C5	0.8682 (3)	−0.0865 (3)	0.3885 (2)	0.0277 (6)	
H5	0.9195	−0.1592	0.3502	0.033*	
C6	0.8655 (3)	0.0716 (3)	0.3651 (2)	0.0272 (6)	
C7	0.9402 (4)	0.1291 (4)	0.2790 (2)	0.0350 (7)	
H7A	0.8549	0.1768	0.2299	0.053*	
H7B	0.9899	0.0401	0.2488	0.053*	
H7C	1.0246	0.2079	0.3017	0.053*	
C8	0.7926 (4)	0.3475 (3)	0.3986 (2)	0.0359 (7)	
H8A	0.8491	0.3661	0.3417	0.054*	
H8B	0.8486	0.4052	0.4562	0.054*	
H8C	0.6789	0.3841	0.3828	0.054*	
C9	0.6423 (4)	0.2444 (3)	0.5574 (2)	0.0333 (7)	
H9A	0.7267	0.3153	0.5913	0.050*	
H9B	0.5878	0.1900	0.6064	0.050*	
H9C	0.5612	0.3059	0.5129	0.050*	
H1	0.758 (4)	−0.306 (4)	0.538 (3)	0.040 (10)*	
H1W	0.344 (5)	0.306 (4)	0.316 (2)	0.059 (12)*	
H2W	0.250 (4)	0.423 (4)	0.319 (3)	0.076 (15)*	

### Atomic displacement parameters (Å^2^)

**Table:**

	*U*^11^	*U*^22^	*U*^33^	*U*^12^	*U*^13^	*U*^23^
Cl1	0.0365 (4)	0.0264 (4)	0.0339 (4)	0.0008 (3)	0.0058 (3)	−0.0006 (3)
O1	0.0452 (12)	0.0180 (9)	0.0385 (12)	0.0037 (8)	0.0100 (10)	0.0015 (8)
O1W	0.0431 (12)	0.0233 (10)	0.0373 (12)	0.0021 (9)	0.0053 (10)	0.0010 (9)
N1	0.0299 (12)	0.0175 (11)	0.0321 (13)	−0.0008 (9)	0.0028 (9)	−0.0003 (9)
C2	0.0268 (13)	0.0220 (12)	0.0278 (14)	0.0005 (10)	−0.0008 (11)	−0.0022 (10)
C3	0.0294 (13)	0.0247 (13)	0.0278 (14)	−0.0006 (11)	0.0023 (11)	−0.0003 (11)
C4	0.0301 (14)	0.0192 (12)	0.0299 (14)	−0.0001 (11)	−0.0002 (11)	−0.0025 (11)
C5	0.0302 (14)	0.0235 (14)	0.0288 (14)	0.0011 (11)	0.0031 (11)	−0.0017 (10)
C6	0.0266 (13)	0.0255 (14)	0.0287 (14)	−0.0020 (10)	0.0015 (11)	−0.0024 (11)
C7	0.0392 (16)	0.0300 (14)	0.0364 (16)	−0.0040 (12)	0.0076 (12)	0.0002 (13)
C8	0.0434 (17)	0.0168 (13)	0.0483 (18)	0.0002 (12)	0.0098 (14)	0.0034 (12)
C9	0.0399 (16)	0.0242 (14)	0.0351 (16)	0.0015 (12)	0.0041 (12)	−0.0051 (12)

### Geometric parameters (Å, º)

**Table:**

O1—C4	1.333 (3)	C5—C6	1.378 (4)
O1—H1	0.82 (4)	C5—H5	0.9500
O1W—H1W	0.812 (19)	C6—C7	1.494 (4)
O1W—H2W	0.824 (19)	C7—H7A	0.9800
N1—C6	1.364 (3)	C7—H7B	0.9800
N1—C2	1.372 (3)	C7—H7C	0.9800
N1—C8	1.484 (3)	C8—H8A	0.9800
C2—C3	1.367 (4)	C8—H8B	0.9800
C2—C9	1.495 (4)	C8—H8C	0.9800
C3—C4	1.396 (4)	C9—H9A	0.9800
C3—H3	0.9500	C9—H9B	0.9800
C4—C5	1.394 (4)	C9—H9C	0.9800
			
C4—O1—H1	107 (2)	C5—C6—C7	120.4 (3)
H1W—O1W—H2W	101 (4)	C6—C7—H7A	109.5
C6—N1—C2	121.0 (2)	C6—C7—H7B	109.5
C6—N1—C8	120.6 (2)	H7A—C7—H7B	109.5
C2—N1—C8	118.4 (2)	C6—C7—H7C	109.5
C3—C2—N1	120.0 (2)	H7A—C7—H7C	109.5
C3—C2—C9	120.9 (2)	H7B—C7—H7C	109.5
N1—C2—C9	119.1 (2)	N1—C8—H8A	109.5
C2—C3—C4	120.4 (3)	N1—C8—H8B	109.5
C2—C3—H3	119.8	H8A—C8—H8B	109.5
C4—C3—H3	119.8	N1—C8—H8C	109.5
O1—C4—C5	118.6 (2)	H8A—C8—H8C	109.5
O1—C4—C3	122.9 (3)	H8B—C8—H8C	109.5
C5—C4—C3	118.5 (2)	C2—C9—H9A	109.5
C6—C5—C4	120.5 (3)	C2—C9—H9B	109.5
C6—C5—H5	119.8	H9A—C9—H9B	109.5
C4—C5—H5	119.8	C2—C9—H9C	109.5
N1—C6—C5	119.7 (3)	H9A—C9—H9C	109.5
N1—C6—C7	119.9 (2)	H9B—C9—H9C	109.5
			
C6—N1—C2—C3	1.8 (4)	O1—C4—C5—C6	−179.4 (3)
C8—N1—C2—C3	−179.5 (3)	C3—C4—C5—C6	0.7 (4)
C6—N1—C2—C9	−179.4 (2)	C2—N1—C6—C5	−2.4 (4)
C8—N1—C2—C9	−0.7 (3)	C8—N1—C6—C5	178.9 (3)
N1—C2—C3—C4	0.1 (4)	C2—N1—C6—C7	177.7 (2)
C9—C2—C3—C4	−178.7 (2)	C8—N1—C6—C7	−1.0 (4)
C2—C3—C4—O1	178.7 (3)	C4—C5—C6—N1	1.2 (4)
C2—C3—C4—C5	−1.3 (4)	C4—C5—C6—C7	−179.0 (2)

### Hydrogen-bond geometry (Å, º)

**Table:**

*D*—H···*A*	*D*—H	H···*A*	*D*···*A*	*D*—H···*A*
O1—H1···O1*W*^i^	0.82 (4)	1.78 (4)	2.591 (3)	168 (4)
O1*W*—H1*W*···Cl1	0.81 (2)	2.31 (2)	3.095 (2)	162 (4)
O1*W*—H2*W*···Cl1^ii^	0.82 (2)	2.30 (2)	3.106 (2)	168 (4)
C3—H3···Cl1^i^	0.95	2.72	3.647 (3)	165
C9—H9*A*···Cl1^iii^	0.98	2.80	3.704 (3)	154

Symmetry codes: (i) −*x*+1, −*y*, −*z*+1; (ii) −*x*+1/2, *y*+1/2, −*z*+1/2; (iii) *x*+1/2, −*y*+1/2, *z*+1/2.
